# Effect of Alpha-Particle Irradiation on InGaP/GaAs/Ge Triple-Junction Solar Cells

**DOI:** 10.3390/ma11060944

**Published:** 2018-06-04

**Authors:** Jing Xu, Min Guo, Ming Lu, Hu He, Guang Yang, Jianwen Xu

**Affiliations:** 1 Department of Physics, Yantai University, 30 Qingquan Road, Laishan District, Yantai 264005, China; xj2012@mail.bnu.edu.cn (J.X.); 17853501729@163.com (M.G.); 2 Shanghai Institute of Space Power-Sources, 2965 Dongchuan Road, Minhang District, Shanghai 200233, China; hh28-121@163.com (H.H.); yg_shuihaozi@sina.com (G.Y.); 13671578748@163.com (J.X.)

**Keywords:** InGaP/GaAs/Ge solar cells, alpha-particle irradiation, current–voltage (I–V) characteristics, spectral response, photoluminescence, degradation

## Abstract

InGaP/GaAs/Ge triple-junction solar cells were irradiated with 5.1 MeV alpha particles with different fluences. The degradations of the optical and electrical properties of InGaP/GaAs/Ge solar cells were described in terms of the variation in the short-circuit current (I_sc_), the open-circuit voltage (V_oc_), the maximum power (P_max_), the spectral response (SR), and the photoluminescence (PL) versus the 5.1 MeV alpha-particle fluences. The degradation modeling of the I_sc_ and V_oc_ under 1 MeV, 3 MeV, and 5.1 MeV alpha-particle irradiation was performed by calculating the introduction rate of non-radiative recombination centers, and the minority-carrier capture cross section, and the results were in good agreement with experimental data. For comparison, the degradations of the I_sc_ and V_oc_ were presented under 1 MeV and 3 MeV proton irradiation.

## 1. Introduction

The near-earth space radiation environment in which solar cells are located mainly consists of electrons and protons trapped in the earth’s radiation belts. Specifically, galactic cosmic rays (GCRs) consist of 87% protons, 12% alpha particles, and 1% heavier ions, while solar particle events (SPEs) consist of 96.4% protons, 3.5% alpha particles, and 0.1% heavier ions [1]. However, this doesn’t mean that the alpha particles and heavier ions in GCRs and SPEs can be ignored despite their small percentages. Protons and alpha particles, as well as heavier ions, degrade the optical and electrical properties of solar cells. In addition, when the particles in GCRs and SPEs travel through the earth’s atmosphere, they interact with the electrons and the nuclei of atoms in the atmosphere, resulting in the production of secondary particles [2]. These secondary particles can also cause the degradation of solar cells. Therefore, it is necessary to study the response of solar cells under different types of particle irradiation in an effort to satisfy the requirements of high reliability and long life for spacecrafts.

For the past few years, the InGaP/GaAs/Ge triple-junction (3J) solar cells became the mainstream generation of space power in spacecraft due to its high conversion efficiency and radiation hardness [3,4,5,6,7,8,9]. However, the studies on the radiation response of InGaP/GaAs/Ge triple-junction solar cells were mostly concentrated on proton and electron irradiation [3,4,5,6,7,8], with few researches focusing on alpha-particle irradiation [9]. In Reference [3], the effects of 10 MeV proton irradiation on InGaP/GaAs/Ge triple-junction solar cells were studied experimentally and theoretically. The results indicated that the damage of the GaAs subcell was the highest, while InGaP and Ge subcells exhibited slight signs of degradation with the same fluence. In Reference [5], the impact of 1 MeV electron irradiation-induced non-radiative recombination centers on each subcell of the InGaP/InGaAs/Ge solar cell was analyzed in detail based on electroluminescence characteristics. The results indicated that the open-circuit voltages of the InGaP and Ge subcells showed only a little degradation, but a significant drop was observable for the InGaAs middle subcell. In fact, although the relative abundance of alpha particles is less than that of protons in SPEs and GCRs, the displacement damage of solar cells induced by alpha particles was more serious than that for protons at the same energy because the non-ionizing energy loss (NIEL) of alpha particles is about 10 times that of protons [10]. Additionally, alpha-particle energy can be as high as 10^14^ MeV and 10^3^ MeV in GCRs and SPEs, respectively [1]. These high-energy alpha particles can travel through the cover glass in the front surface of the solar cells, and fall on the active regions of the cells, causing the degradation of their optical and electrical properties.

In our previous work, we studied the radiation effects on InGaP/GaAs/Ge solar cells under 0.32–3 MeV proton irradiation and under 1–11.5 MeV electron irradiation [7,8]. On this basis, we firstly analyzed the degradation of the optical and electrical properties of InGaP/GaAs/Ge solar cells under 5.1 MeV alpha-particle irradiation based on current–voltage (I–V) characteristics, spectral response (SR), and photoluminescence (PL) measurements. We then simulated the degradation of the short-circuit current (I_sc_) and the open-circuit voltage (V_oc_) under 1 MeV, 3 MeV, and 5.1 MeV alpha-particle irradiation by calculating the introduction rate of non-radiative recombination centers, and the minority-carrier capture cross section. Finally, we compared the degradation behaviors of I_sc_ and V_oc_ under 1 MeV and 3 MeV alpha-particle and proton irradiation.

## 2. Materials and Methods 

The InGaP/GaAs/Ge 3J solar cells were fabricated on Ge substrates by metalorganic chemical vapor deposition. The solar cells consisted of InGaP top cells, GaAs middle cells, and Ge bottom cells. The thicknesses of the InGaP, GaAs, and Ge subcells were about 1.2 µm, 3 µm, and 1 µm, respectively. Each subcell was connected by a tunnel junction. The detailed structures of the solar cells are shown in Reference [11] (p. 746).

The InGaP/GaAs/Ge 3J solar cells were irradiated by alpha particles liberated from a ^239^Pu surface source. Five ^239^Pu alpha sources were chosen for the irradiation experiment, and all sources were disc-shaped with a diameter (*d*) of 2.5 cm. The total activity (*A*) of the ^239^Pu source, as well as the irradiation time (*t*), is listed in Table 1. The fluences (*φ*) were expressed as $\varphi =4At/\pi d^{2}$, and its values are listed in Table 1. During the decay of ^239^Pu to ^235^U, 70.8% of the alpha particles were emitted with an energy which peaked sharply at 5.157 MeV, while 17.1% had an energy of 5.144 MeV, and 11.9% had an energy of 5.105 MeV [12]. The alpha particles of these three energies (5.157 MeV, 5.144 MeV, and 5.105 MeV) accounted for 99.8% of all the alpha particles liberated from the ^239^Pu source, and their values were very close. Therefore, the alpha particles liberated from the ^239^Pu source were approximately considered as 5.1 MeV monoenergetic particles in this paper. The experiment was performed in the air. During the experiment, the solar cells with no cover glass were attached tightly to the surface of the ^239^Pu sources. It was assumed that the thickness of the air layer between the ^239^Pu source and the solar cell was negligibly small. Therefore, we considered that there was almost no alpha-particle energy loss in the area between the Pu source and the solar cell.

The I–V characteristics of the InGaP/GaAs/Ge 3J solar cells before and after alpha-particle irradiation were measured under Air Mass zero (AM0) using a solar simulator with an illumination of 136.7 mW·cm^−2^. The I_sc_, V_oc_, and maximum power (P_max_) were extracted from the I–V measurements. The SR of the InGaP top cells and GaAs middle cells were measured before and after alpha-particle irradiation in various bias-light conditions. The PL spectra of the InGaP top cells and GaAs middle cells were measured before and after alpha-particle irradiation by a 532 nm and a 730 nm laser, respectively.

## 3. Results and Discussion

### 3.1. The Distribution of the Displacement Damage in Solar Cells

**Figure 1:**
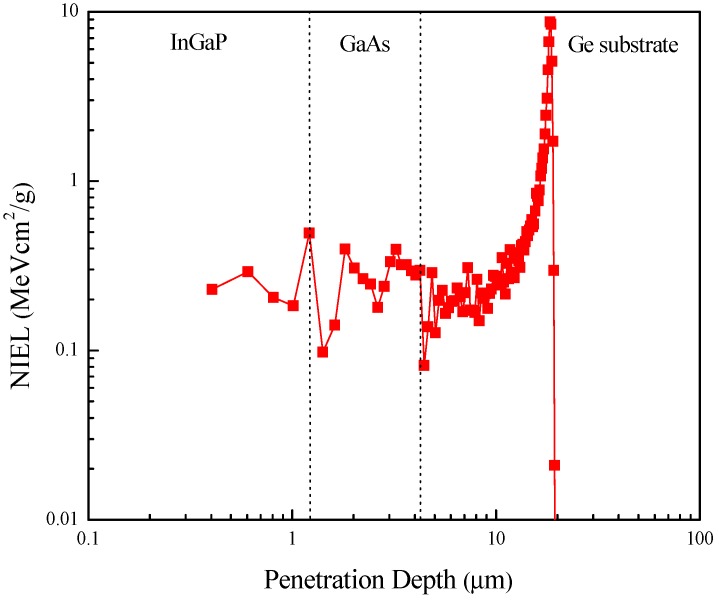
Non-ionizing energy loss (NIEL) as a function of 5.1 MeV alpha-particle penetration depth in the InGaP/GaAs/Ge solar cells.

For the displacement-damage estimation, the non-ionizing energy loss (NIEL) gave the portion of energy lost due to displacement damage. The NIEL for the 5.1 MeV alpha particles in the InGaP/GaAs/Ge 3J solar cells was simulated by the Stopping and Range of Ions in Matter (SRIM) [13], and is shown in Figure 1. The displacement threshold energies used in the simulation were 10 eV for both Ga and As, 6.7 eV for In, 8.7 eV for P, and 27 eV for Ge [11]. In Figure 1, the Bragg damage peak of the 5.1 MeV alpha particles occurred at the end of the track at a depth of 11 µm, and was far away from the major active region of the three subcells. Thus, it was assumed that the distribution of displacement damage in the three subcells was uniform, and that the NIEL ≈$0.3\;\mathrm{MeV}\cdot {\mathrm{cm}}^{2}\cdot g^{-1}$ for the 5.1 MeV alpha particles.

### 3.2. I–V Characteristics before and after Alpha-Particle Irradiation

**Figure 2:**
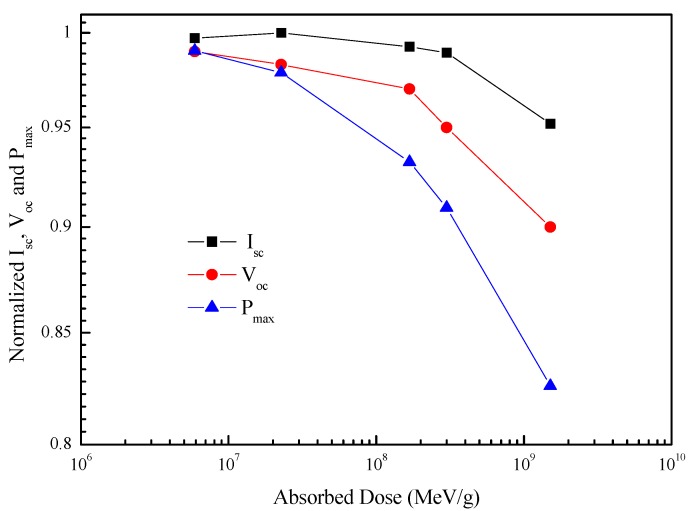
Normalized short-circuit current (I_sc_), open-circuit voltage (V_oc_), and the maximum power (P_max_) in the InGaP/GaAs/Ge triple-junction (3J) solar cells as a function of the absorbed dose.

The absorbed dose, which is a product of energy deposited by ionizing radiation in a certain mass of material, can be calculated by multiplying alpha-particle fluence and NIEL. Figure 2 shows the degradation trends of I_sc_, V_oc_, and P_max_ in InGaP/GaAs/Ge solar cells as a function of the absorbed dose.

The degradation rates of the I_sc_, V_oc_, and P_max_ increased with an increase in absorbed dose. The primary reason for the degradation of I_sc_, V_oc_, and P_max_ is the reduction of the minority-carrier lifetime due to alpha-particle radiation-induced recombination centers in solar cells. As seen in Figure 2, P_max_ degradation was the most remarkable, as P_max_ is proportional to the product of the current and the voltage. The I_sc_ degraded less than the V_oc_, because V_oc_ degradation was contributed to by the sum of all three subcells, while I_sc_ degradation was only contributed to by one of the three subcells.

### 3.3. The SR before and after Alpha-Particle Irradiation 

**Figure 3:**
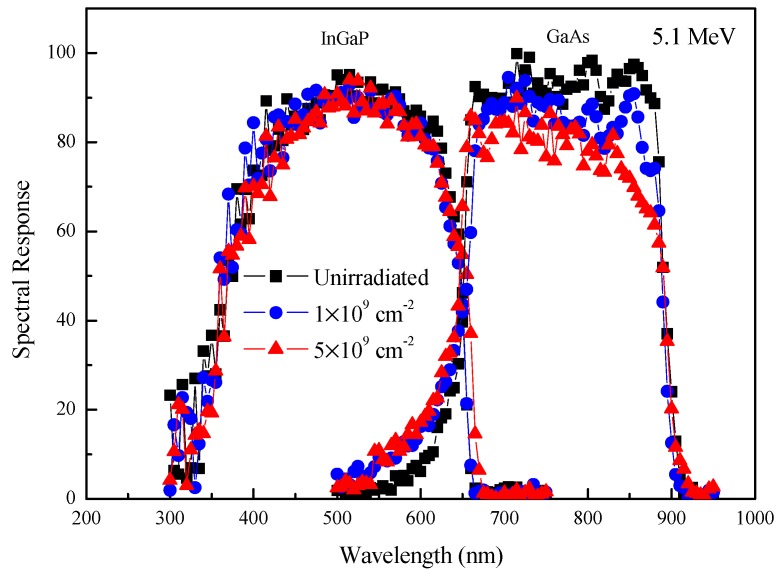
Spectral response of the InGaP top cells and GaAs middle cells before and after 5.1 MeV alpha-particle irradiation with various fluences.

Figure 3 shows the changes in SR of the InGaP top cells and GaAs middle cells before and after 5.1 MeV alpha-particle irradiation with fluences of 1 × 10^9^ cm^−2^ and 5 × 10^9^ cm^−2^. The SR of the Ge bottom cells was not plotted in Figure 3 because the Ge subcells had a big-current and a small-voltage contribution to the InGaP/GaAs/Ge 3J solar cells [7]. In Figure 3, it can be noted that the SR dropped at wavelengths ranging from 650 nm to 900 nm for the GaAs middle cells after alpha-particle irradiation. However, the SR hardly decreased at wavelengths ranging from 350 nm to 650 nm for the InGaP top cells. This was mainly because there was lower migration energy of In (0.26 eV) and P (1.2 eV) in InGaP when compared with that of Ga (1.79 eV) and As (1.48 eV) in GaAs [14]. This meant that the GaAs middle cells were less radiation-resistant than the InGaP top cells. For InGaP/GaAs/Ge 3J solar cells, the photovoltage is the sum of the voltage for all three subcells, while the photocurrent is limited to the smallest value for any of the three subcells [15]. As a result, the degradation of the InGaP/GaAs/Ge solar cells was primarily controlled by the most radiation-sensitive subcell. Therefore, according to the SR measurements in Figure 3, the degradation of the InGaP/GaAs/Ge 3J solar cells was primarily controlled by the GaAs middle cells after alpha-particle irradiation.

### 3.4. The PL Spectra before and after Alpha-Particle Irradiation 

**Figure 4:**
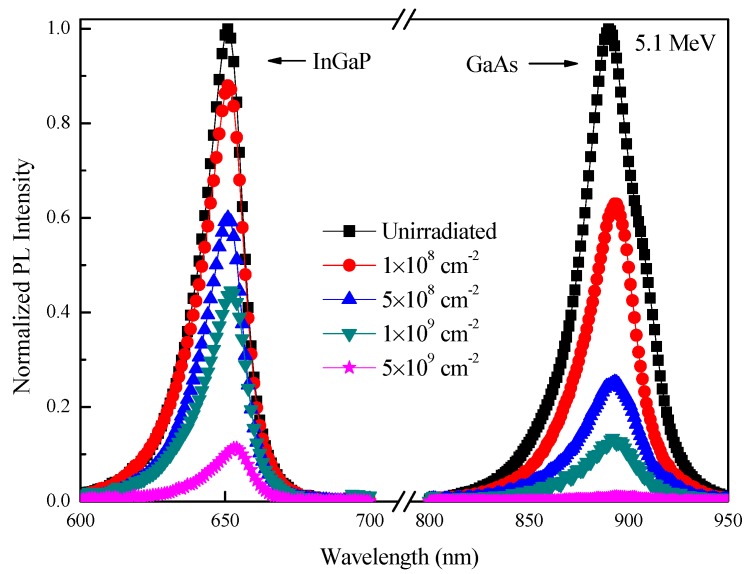
Photoluminescence (PL) spectra at room temperature of the InGaP top cells and GaAs middle cells before and after alpha-particle irradiation with various fluences.

Figure 4 shows the changes in PL spectra of the InGaP top cells and GaAs middle cells at room temperature before and after 5.1 MeV alpha-particle irradiation with various fluences. As in Section 3.3, the PL spectra of the Ge bottom cells were not plotted in Figure 4. The PL intensity of the InGaP top cells and GaAs middle cells decreased with an increase in alpha-particle fluence, but the PL degradation of the GaAs middle cells was more than that of the InGaP top cells. This further corroborated that the GaAs middle cells were less radiation-resistant than the InGaP top cells.

**Figure 5:**
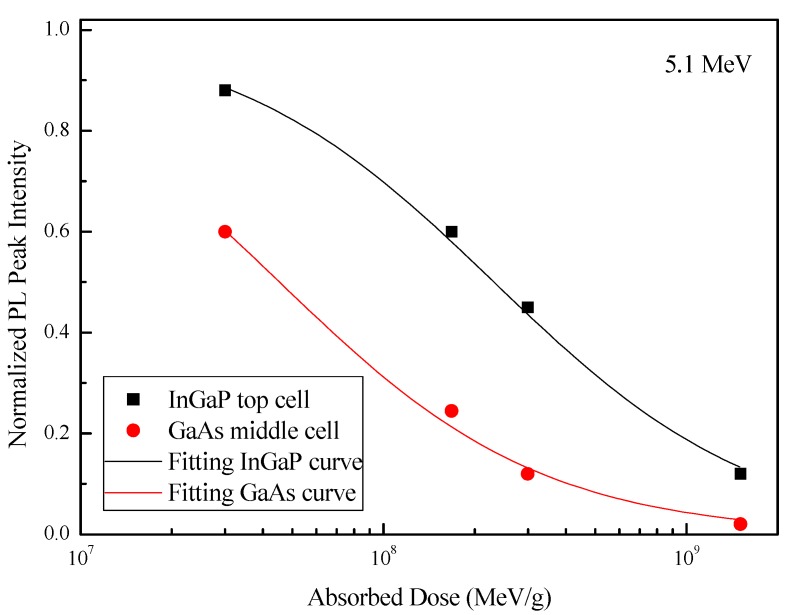
Normalized PL peak intensity of the InGaP top cells and GaAs middle cells as a function of the absorbed dose.

Figure 5 shows the changes in normalized PL peak intensity of the InGaP top cells and GaAs middle cells after alpha-particle irradiation with an absorbed dose ranging from 3 × 10^7^ MeV/g to 1.5 × 10^9^ MeV/g. The relationship between PL peak intensity (η) and the absorbed dose (D) can be expressed as [16]

(1) $$\eta ={\left(1+\frac{\alpha }{\mathrm{NIEL}}D\right)}^{-1},\;$$

where α=kσν/BN, and k is the introduction rate of the non-radiative recombination centers, σ is the minority-carrier capture cross section, ν is the thermal velocity of the carriers, B is the radiative recombination probability, and N is the doping concentration. The values of ν, B, and N are listed in Table 2.

As shown in Figure 5, the fitting curve obtained by Relationship (1) agreed with experimental data. The fitting parameters (α) are listed in Table 2. The values of kσ were calculated, and are also listed in Table 2. Next, the values of kσ for the GaAs middle cell were used for modeling the degradation of the InGaP/GaAs/Ge 3J solar cells, which was primarily controlled by the GaAs middle cell after alpha-particle irradiation.

### 3.5. Degradation Modeling of the Solar Cells

**Figure 6:**
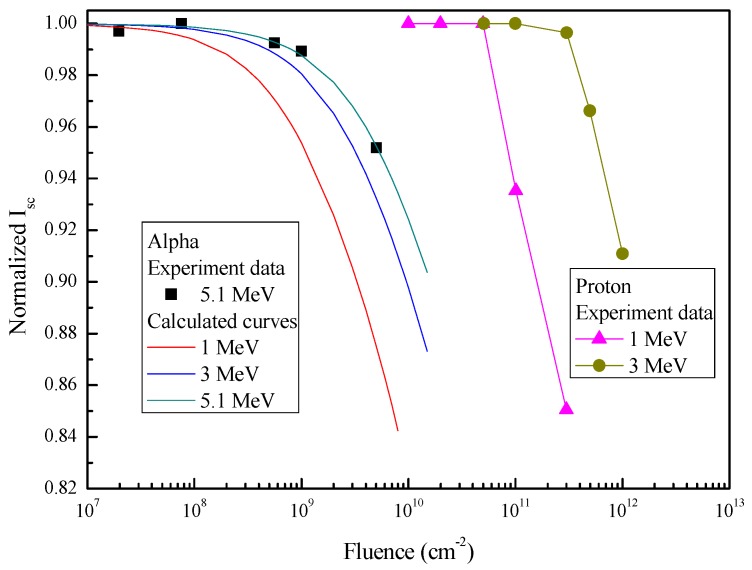
Normalized I_sc_ of the InGaP/GaAs/Ge solar cells as a function of the fluence for various alpha-particle and proton energies.

**Figure 7:**
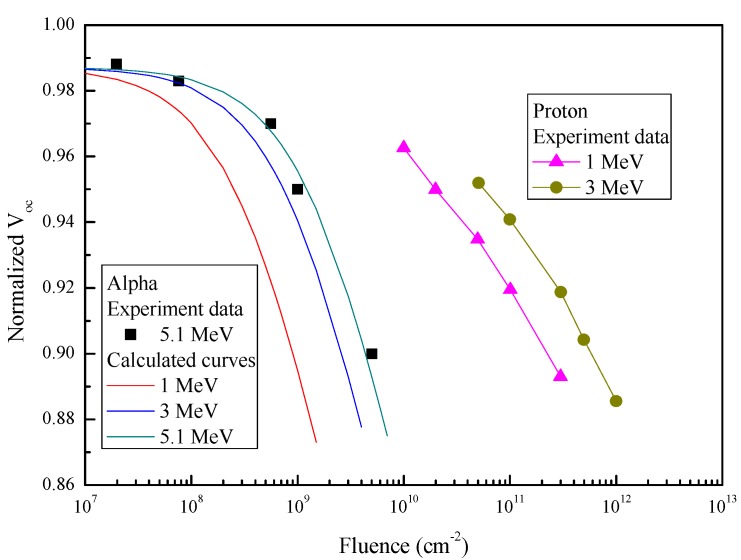
Normalized V_oc_ of the InGaP/GaAs/Ge solar cells as a function of the fluence for various alpha-particle and proton energies.

Degradation modeling of the InGaP/GaAs/Ge 3J solar cells under alpha-particle irradiation with various energies and fluences was performed using a theoretical method [17,18]. This approach was introduced in detail in our previous work [18]. In this method, we considered that the main reason for the degradation of the solar cells was the non-radiative recombination centers induced by alpha-particle irradiation in the active regions of the cells. Therefore, according to the kσ value for the GaAs cells in Table 2, the degradation curves for I_sc_ and V_oc_ as a function of the fluence under 5.1 MeV alpha-particle irradiation were obtained, and are shown in Figure 6 and Figure 7, respectively. The degradation curves showed good agreement with experimental data. For modeling the degradation of solar cells under various alpha-particle energies, the kσ values can be calculated by the following relationship [17]:

(2) kσ(E)=βNIEL(E),

where *E* is the incident alpha-particle energy, β is a constant, and NIEL is the non-ionizing energy loss. For 5.1 MeV alpha particles in the GaAs material, $\mathrm{NIEL}=0.3\;\mathrm{MeV}\cdot {\mathrm{cm}}^{2}\cdot g^{-1}$ [19], and $k\sigma =1.2\times 10^{-10}\;\mathrm{cm}$ (see Table 2). Therefore, according to Relationship (2), $\beta =4\times 10^{-10}\;g\cdot {\mathrm{MeV}}^{-1}\cdot {\mathrm{cm}}^{-3}$. As an example, 1 MeV and 3 MeV alpha particles were selected, and their NIEL is listed in Table 3. The values of kσ for 1 MeV and 3 MeV alpha particles were calculated using Relationship (2), and are also listed in Table 3.

According to the kσ values in Table 3, the degradations of I_sc_ and V_oc_ for 1 MeV and 3 MeV alpha-particle irradiation were predicted, and are shown in Figure 6 and Figure 7, respectively. Meanwhile, the degradation data of I_sc_ and V_oc_ for 1 MeV and 3 MeV proton irradiation are also shown in Figure 6 and Figure 7, respectively. As seen in both figures, the fluence of alpha-particle irradiation was about two orders of magnitude lower than that of proton irradiation for the same energy, when the degradation was identical. This was mainly because the introduction rate of defects for alpha particles is much larger than that for protons [20]. This meant that, although the relative abundance of alpha particles is lower than that of protons in the space environment, alpha particles cause a more serious degradation of space solar cells than that due to protons. 

## 4. Conclusions

The effects of 5.1 MeV alpha-particle irradiation on InGaP/GaAs/Ge 3J solar cells were studied in detail using I–V characteristics, and measurements of SR and PL. Alpha particles had influence on the GaAs subcell, while having less influence on the InGaP subcell. The products of kσ for 1 MeV, 3 MeV, and 5.1 MeV alpha particles were calculated by means of PL spectra and NIEL. The degradations of I_sc_ and V_oc_ under 1 MeV, 3 MeV, and 5.1 MeV alpha-particle irradiation were simulated, and the results were in good agreement with experimental data. In summary, alpha particles in the space environment cannot be ignored, as they can cause a more serious degradation of space solar cells than that due to protons.

## Figures and Tables

**Table 1:** The values of irradiation time (*t*), fluence (*φ*), and total activity (*A*).

Parameters	Source 1	Source 2	Source 3	Source 4	Source 5
*A* (Bq)	1.20 × 10^2^	4.23 × 10^2^	3.47 × 10^3^	1.03 × 10^3^	3.20 × 10^3^
*t* (h)	227	247	218	1360	2130
φ (cm^−2^)	2.0 × 10^7^	7.6 ×10^7^	5.6 × 10^8^	1.0 × 10^9^	5.0 × 10^9^

**Table 2:** Values of α, ν, B, N, and kσ for 5.1 MeV alpha-particle irradiation in the InGaP and GaAs subcells.

Cells	α (cm^2^)	ν (cm/s)	B ($cm^{3}\cdot s^{-1}$)	N (cm^−3^)	kσ (cm)
InGaP	1.3 × 10^−9^	3.5 × 10^7^	2 × 10^−10^	1 × 10^16^	7.4 × 10^−11^
GaAs	6.6 × 10^−9^	5 × 10^7^	1.5 × 10^−10^	6 × 10^15^	1.2 × 10^−10^

**Table 3:** Values of non-ionizing energy loss (NIEL) and kσ for 1 MeV and 3 MeV alpha particles in the GaAs material.

Alpha-Particle Energy (MeV)	NIEL ($MeV\cdot cm^{2}\cdot g^{-1}$)	kσ (cm)
1	1.5 ^1^	6 $\times 10^{-10}$
3	0.5 ^1^	2 $\times 10^{-10}$

^1^ Reference [19].
